# Acquisition of Incidental Bidirectional Naming: Isolating the Effects of Probing and Mixed-Operant Instruction

**DOI:** 10.1007/s40616-025-00221-1

**Published:** 2025-08-07

**Authors:** Heidi Skorge Olaff, Per Holth

**Affiliations:** Faculty of Health Science, Institute of Behavioural Science, OsloMet––Oslo Metropolitan University, Post Box 4, St. Olavs Plass, 0130 Oslo, Norway

**Keywords:** Autism, Incidental bidirectional naming, Inc-BiN, Mixed-operant instruction, Repeated probing

## Abstract

The primary purpose of the present experiment was to explore the extent to which repeated probing contributes to the establishment of incidental bidirectional naming (Inc-BiN). Whenever repetitive probes alone did not suffice to establish Inc-BiN, we investigated whether mixed-operant instuction (MOI)––the rapid rotation of operants within each of a series of trial blocks––improved Inc-BiN. Nine children with autism or language delays aged 3–6 participated. Three of nine participants were exposed to an extended-baseline condition, while the remaining six were exposed to one of two brief-baseline conditions before MOI. We used a multiple probe design across three novel stimulus sets, to isolate the effects of repeated probing. During post-MOI Inc-BiN probes, all participants across conditions demonstrated the emergence of Inc-BiN. Repetitive probes sufficed to establish Inc-BiN in two of three participants who were assigned to the extended-baseline condition, while for the third, Inc-BiN improved after MOI. In addition, we examined the extent to which the probe sequence impacted Inc-BiN skills. Three participants, P1, P2, and P3, were exposed to speaker (tacts) probes first, while the remaining six were exposed to listener probes first. During generative Inc-BiN probes, when testing speaker responses before listener responses (P1–P3), only listener responses emerged for two of them. In contrast, when testing listener before speaker responses, both repertoires were observed for three (P4, P5, and P7) of six participants. A one-month follow-up Inc-BiN probe demonstrated maintenance of listener responses for seven of eight participants, and tacts were maintained for three of them.

Naming has been characterized as the joining or integration of the listener and speaker repertoires in human behavior (e.g., Greer & Longano, 2010; Greer & Speckman, 2009; Horne & Lowe, 1997; Miguel, 2016, 2018). Listener responses included in naming are pointing to, orienting toward, or otherwise selecting an object or event when asked to do so. The speaker operants include tacts (i.e., saying the name of a stimulus in its presence) and echoics (i.e., vocal imitation). To distinguish the concept of naming from merely labeling or tacting, Miguel (2016, 2018) suggested bidirectional naming (BiN) as a less ambiguous term, which we adopt here.

A distinction has been made between common bidirectional naming (C-BiN) and incidental bidirectional naming (Inc-BiN). C-BiN is evident if training of listener responses leads to the emergence of speaker responses, or vice versa (Miguel, 2016). In Inc-BiN (Hawkins et al., 2018), both listener and speaker responses are emitted by the individual following another individual’s tact of a novel stimulus. For example, a caregiver says, “Look, what a beautiful sapphire” while pointing to it. Out of such an incidental episode, referred to as a naming experience, the child may subsequently (1) respond as a listener by orienting or pointing to the sapphire when a caregiver asks, “Where is the sapphire?”, (2) say “sapphire” by vocally repeating the caregiver’s tact (i.e., echoic), (3) on following occasions say “sapphire” in its presence (i.e., tact) or when asked “What is this?” in the presence of a sapphire. In accord with Skinner’s (1957) terminology, we refer to the latter case as a manded tact. Thus, Inc-BiN consists of echoics, tacts, manded tacts, and listener responses, as well as joint attention skills (e.g., Catania, 2013, p. 319; Gilmore et al., 2024). The joint attention skills include pointing or orienting toward the sapphire when asked where it is (responding to joint attention) and directing others’ attention to the sapphire by pointing or looking toward it (initiating joint attention). Joint attention is a critical prerequisite skill for Inc-BiN skills because these skills are not just responding in *the presence of* objects or events but responding *under control* of specific objects or events. Before the implementation of an intervention to facilitate Inc-BiN, these prerequsites may need to be established separately.

Inc-BiN is an important prerequisite for a range of other essential skills. Researchers have demonstrated that the procedures that lead to the experimental demonstration of Inc-BiN also increase language development and symbolic verbal behavior more generally (e.g., Greer & Keohane, 2005; Horne & Lowe, 1996), as well as stimulus categorization skills (Horne et al., 2004; Kobari-Wright & Miguel, 2014; Lechago et al., 2015; Lowe et al., 2002, 2005; Miguel & Kobari-Wright, 2013; Miguel et al., 2008; Petursdottir et al., 2008). In addition, the contingencies responsible for Inc-BiN lead to “the rapid vocabulary growth of 3-year-old children” (Gilic & Greer, 2011, p. 158) and “tacting with understanding” (Miguel, 2016, p. 129). Moreover, Inc-BiN procedures have led to representational drawing skills (i.e., drawing objects that the participant has observed; Dixon et al., 2017), reading with understanding (Horne & Lowe, 1996; LaFrance & Miguel, 2014, p. 16), spelling and writing (e.g., Eby et al., 2010), faster learning of complex language skills (Greer & Du, 2015), and faster learning from observing models (Greer et al., 2011). Greer et al. demonstrated that the participants who lacked Inc-BiN required extensive instruction to achieve educational objectives. Furthermore, Hranchuk et al. (2019) found that children who engaged in Inc-BiN learned more than twice as fast (i.e., required fewer trials) as childen who did not engage in Inc-BiN. Because children with Inc-BiN skills appear to acquire responding more efficiently and in new ways and are able to learn targets that they could not before these skills were established, Inc-BiN is considered a behavioral cusp (e.g., Greer & Longano, 2010; Sivaraman et al., 2023). Unfortunately, children with autism often do not engage in Inc-BiN (e.g., Greer & Longano, 2010; Grow & Kodak, 2010; Sivaraman et al., 2021). Given the critical nature of this skill, it behooves us to develop ways to teach Inc-BiN to individuals with autism and any others who do not demonstrate it.

Multiple-exemplar instruction has been identified as a successful intervention for the production of Inc-BiN skills in children with autism or language delays (e.g., Byrne et al., 2014; Gilic & Greer, 2011; Greer et al., 2005, 2007; Hawkins et al., 2009; Morgan et al., 2021; Olaff et al., 2017; Yoon et al., 2023). Multiple-exemplar instruction involves rotating or intermixing different operants across several members of a set of stimuli. Typically, the operants that are intermixed include (1) selection responses during (auditory + visual)-visual matching-to-sample (MTS) and (2) auditory-visual MTS (i.e., listener responses), as well as (3) tacts and (4) manded tacts (i.e., speaker responses). We will use the terms mixed-operant instruction (MOI; cited in Cooper et al., 2020, p. 810; LaFrance et al., 2019) to emphasize that different operants (e.g., pointing to a cat, tacting cat, and, possibly, echoing “cat”) are rapidly rotated across trials involving the same stimulus. A detailed description of MOI is included in the experimental procedure below.

It is common for children to learn through incidental exposure to novel names of objects in the natural environment, by observing a caregiver uttering the object’s name (e.g., Horne & Lowe, 1996; Moerk, 1990). A demonstration of the emergence of Inc-BiN skills requires, in one way or another, an exposure to relevant novel stimuli while simultanously hearing the names of those stimuli (i.e., a naming experience) before Inc-BiN is probed (e.g., Cao & Greer, 2019; Gilmore et al., 2024; Greer & Longano, 2010; Morgan et al., 2021; Sivaraman et al., 2023). The naming experience, often used in MOI studies (e.g., Gilic & Greer, 2011; Greer et al., 2005, 2007; Lee et al., 2021; Olaff et al., 2017), consists of simultaneous visual-visual MTS tasks in which the researcher states the name of the sample stimulus––(auditory + visual)-visual MTS––without response requirements beyond a selection response, such as echoing (e.g., Cao & Greer, 2019; Gilic & Greer, 2011; Greer & Ross, 2008; Greer et al., 2007; Hotchkiss & Fienup, 2020). Whether or not an echoic response is required during a naming experience, the reinforcement procedures are the same: correct selection responses are differentially reinforced until mastery, and incorrect responses are usually followed by an error correction and prompting procedure. Next, the Inc-BiN probes are carried out with the same stimuli as used during the naming experience, but without programmed differential consequences. The Inc-BiN probes test each operant separately. Inc-BiN skills are evident by the number of listener responses, tacts, and/or manded tacts. Nevertheless, there is a gap in the research literature regarding whether a naming experience, such as simple exposure to novel names in the presence of specific objects (e.g., Miller et al., 2021) or MTS with the researcher’s tacts of novel stimuli (e.g., Gilic & Greer, 2011) prior to Inc-BiN probes, can enhance Inc-BiN skills (e.g., Petursdottir & Carr, 2011; Sivaraman et al., 2021). Therefore, an important research question is whether it is possible to separate the effects of MOI from the effects of the exposure to a MTS-naming experience.

Furthermore, the test sequence of Inc-BiN probes usually entails first testing pointing to experimenter-tacted objects (i.e., listener response), then tacts and, finally, manded tacts (e.g., Cao & Greer, 2019; Gilic & Greer, 2011; Greer et al., 2007; Longano & Greer, 2015). Testing listener before speaker responses offers the participants an additional opportunity to observe the researcher’s tact, while they are responding to the corresponding stimulus as a listener––before speaker responses are probed. Moreover, the listener probe (e.g., “Point to the okapi”) likely prompt echoing the researcher’s tact (e.g., “okapi”) that may impact the occurrence of tact response (e.g., saying “okapi” in the presence of an image of an okapi). Therefore, during Inc-BiN probes, recent studies provided an alternative test sequence by probing speaker before listener responses (Lee et al., 2021; Olaff et al., 2017).

Regardless of test sequence, recent research has demonstrated Inc-BiN skills in children with autism after exposing participants to visual stimuli while the experimenter states their corresponding names, such as during pairing (e.g., Carnerero & Pèrez-Gonzàlez, 2014; Pérez-González et al., 2014), stimulus pairing observation (e.g., Solares & Fryling, 2019), and in typically developing children during train-test sessions (Petursdottir et al., 2020). Moreover, Kleinert-Ventresca et al. (2023) showed that Inc-BiN skills improved in first-graders, who already demonstrated the listener part of Inc-BiN, following repeated exposure to unfamiliar stimuli. These studies present visual stimuli paired with corresponding experimenter-stated vocal names, and the results suggest that the exposure itself improves Inc-BiN skills (e.g., Kleinert-Ventresca et al., 2023; Petursdottir et al., 2020; Solares & Fryling, 2019).

Sivaraman et al. (2021) exposed eight typically developing young children (*M* age = 18.7 months) to a naming experience, in which auditory and visual stimuli were presented non-simultaneously across trials. The object was first presented to the child, then hidden before it was tacted by the experimenter. Four children received repeated probing only (i.e., four probes), while the other four were exposed to multiple exemplar training (MET) after four probes. Unidirectional naming (UniN; the listener part of Inc-BiN, such as point-to responses; Hawkins et al., 2018) was demonstrated in all four participants following MET, whereas speaker responses emerged in only one of them. When only exposed to stimuli and their names before probing, none of the participants showed consistent improvements in their listener or speaker performances.

The potential effect on the emergence of Inc-BiN from repeated probing using MTS-naming experiences alone has not yet, to our knowledge, been explored. Therefore, it is important to clarify whether repeated probing alone, using the MTS-naming experience, can improve Inc-BiN. The primary pupose of the present study was to explore the extent to which repetitive Inc-BiN probes alone may improve Inc-BiN skills. A second purpose was to investigate whether MOI resulted in the acquisition of Inc-BiN if, or when, repetitive probes did not suffice to establish Inc-BiN. A third purpose was to evaluate whether the probe sequence affected the number of listener and speaker responses (tacts) during Inc-BiN probes, and a fourth purpose to assess the extent to which the emerged Inc-BiN skills were maintained one month after the independent variables were withdrawn.

## Method

### Participants

Nine boys (P1–P9), aged 3.2–6.2 years (*M* = 4.8 years), diagnosed with autism spectrum disorder (ASD), language delays, or intellectual disability participated in the present experiment. Seven participants with ASD (P1, P2, P3, P5, P6, P7, and P8) received 18–25 h of early intensive behavioral intervention per week. The two remaining participants with language delays (P4) and intellectual disability (P9) received individual educational sessions approximately 10 h weekly––consisting of language training involving increasing their vocabulary and language comprehension (i.e., listener responses). All participants were monolingual (Norwegian). However, four of them were exposed to a second language at home (i.e., P4 Iranian, P5 Somali, P6 Syrian, and P7 Somali).

Before the experiment was initiated, the caregivers provided informed consent and the study was approved by the Norwegian Agency for Shared Services in Education and Research. Inclusion criteria for the present study included prerequisites for Inc-BiN: (1) spontaneous echoing of words, (2) visual-visual MTS responses, (3) at least 20 different tacts, (4) 20 manded tacts, and (5) 20 listener responses––to everyday objects, actions, body parts, animals, functions, peers, and relatives (Catania, 2013; Horne & Lowe, 1996; Miguel, 2016; Sivaraman & Barnes-Holmes, 2023; Yoon et al., 2023). The prerequisites were assessed using the Assessment of Basic Language and Learning Skills-Revised (ABLLS-R; Partington, 2006; Appendix A). The results of the ABLLS-R show that the participants’ scores ranged from 85 to 100% mastery in the domain cooperation and reinforcement effectiveness and ranged from 49 to 100% mastery on visual performance, indicating that all participants matched identical visual stimuli. In the domain of receptive language (i.e., listener responses) and labeling (i.e., tacts), the participants obtained scores ranging from 63 to 98% and from 44 to 97% mastery, respectively, which means that they were able to tact and respond as a listener far above the inclusion criterion for the present experiment. Further, the participants’ scores on vocal imitation (i.e., echoic) ranged from 52 to 100% mastery, indicating that all participants echoed words spontaneously. Finally, all participants were able to emit short sentences and scores ranged from 27 to 82% mastery, and they were able to request preferred items and avoid non-preferred stimuli (i.e., mands) within the range of 53% and 96% mastery. Detailed scores on ABLLS-R and characteristics of each participant are provided in Appendix A.

### Setting and Materials

The experiment was conducted in the participants’ teaching rooms in their daycare centers. The teaching rooms differed across participants as they attended different daycare centers. However, P5, P7 and P9 attended the same daycare center but were taught in different teaching rooms. Similarly, P6 and P8 attended the same daycare center and shared the same teaching room. All teaching rooms were approximately 3 × 2 m and were equipped with a shelf with books, bins with materials for each participant’s teaching program, preferred toys, and bins for toys used during breaks. The experimenter (the first author) also brought a binder with materials used during the experiment, a medium-sized suitcase, and a bag with preferred toys, including a tablet with games which served as positive consequences during instructional conditions (see preference assessment below). The teaching rooms included an adult-size table with one chair at each side of the table. The participants used adjustable chairs (e.g., Stokke trip-trap chairs), which were adjusted for a good working position for each child at the table. A third chair was placed in a corner for the observer (a staff member employed at the daycare centers). Only the participant, the experimenter, and a staff member at the daycare center were present in the teaching room. In addition, we used a video camera to record trial blocks for reliability and treatment integrity assessments.

The primary materials were laminated photos (7 × 5 cm) of unfamiliar stimuli for each participant. The photos were individualized for each participant. All stimuli were selected from categories of everyday objects (e.g., vegetables, sport-related items, and flags). The sequence of novel stimulus sets was randomly assigned across Inc-BiN probe blocks and MOI blocks (c.f. Cariveau et al., 2021, 2022) by blindly drawing envelopes with the images of the relevant sets in an effort to reduce experimenter bias (e.g., Ledford et al., 2019).

Each set consisted of five novel stimuli. Up to eight sets were identified for each participant (see Pre-experimental Procedures), for a total of 40 novel stimuli. All stimuli identified within each set for each participant are displayed in Table 1. Sets 1–3 were baseline Inc-BiN probe sets and were used in baseline and post-MOI, when applicable. Sets 4–6 were MOI sets, Set 7 was designated for generative Inc-BiN probes, and Set 8 was used to conduct follow-up Inc-BiN probes to evaluate the maintenance of Inc-BiN skills.

**Table 1:** Overview of stimuli used in the experiment

Participants	Baseline sets	MOI sets	Generative probe sets	Follow-up probe sets
P1	1. Board Games	4. Dog Breeds	7. Sport Items	8. Boats
	Monopoly	Rottweiler	Weights	Fishing boat
	Chess	Dachshund	Badminton	Worm
	Guess who?	Labrador	Go-kart	Cargo
	Snakes & Ladders	Bloodhound	Table Tennis	Kayak
	Ludo	Border collie	Long-ball	Ferry
	2. Berries	5. School Items		
	Blackberries	Gym bag		
	Blueberries	Notebook		
	Blackcurrant	Sharpener		
	Cherries	Penal		
	Cloudberries	Ruler		
	3. Celebrities	6. Flowers		
	Petter Northug	Oxeye daisy		
	Rihanna	Tulips		
	The Crown prince	Anemones		
	The Crown princess	Clover flower		
	Alex (Rosen)	Lily		
P2	1. Sea Related Items	4. Bugs	7. Rare Animals	Not Completed
	Flat fish	Beetle	Meer Kat	
	Jellyfish	Dragonfly	Lemur	
	Scallops	Bumblebee	Hyena	
	Mussels	Tarantella	Weasel	
	Glass jellyfish	Grasshopper	Hamster	
	2. Farm Related Items	5. Vegetables		
	Barn	Peas		
	Hay balls	Broccoli		
	Storehouse	Rutabaga		
	Silo	Asparagus		
	Grain field	Onion		
	3. Flags	6. Constructions		
	Japan	Loader		
	Spain	Lift		
	Germany	Snow plough		
	Brazil	Waste truck		
	Canada	Lawnmower		
P3	1. Star Signs	4. Flowers	5. Celebrities	6. Planets
	Virgo	Lilac	King Harald	Jupiter
	Cancer	Lily	Shave	Pluto
	Libra	Tulip	Rhianna	Venus
	Sagittarius	Oxeye daisy	Mette Marit	Neptune
	Taurus	Clover	Queen Sonja	Saturn
	2. Cities in Europe			
	Berlin			
	Rome			
	Oslo			
	Skopje			
	Amsterdam			
	3. Flags			
	Great Britain			
	Brazil			
	Japan			
	USA			
	China			
P4	1. Flags	4. Fruits	7. Vegetables	8. Sea Related Items
	Japan	Papaya	Leeks	Snail
	USA	Pineapple	Asparagus	Flounder
	Spain	Peach	Beans	Jelly fish
	Brazil	Mango	Onion	Scallops
	Canada	Olive	Celery	Mussels
	2. Board Game	5. Rare Animals		
	Quips	Wolf		
	Chess	Walrus		
	Yahtzee	Wolverine		
	Monopoly	Musk oxen		
	Uno	Badger		
	3. Berries	6. Farm Related Items		
	Lingonberry	Silo		
	Cloudberry	Hay		
	Currant	Barn		
	Cherry	Plow		
	Blackcurrant	Storehouse		
P5	1. Farm Related Items	4. Tools	7. Celebrities	8. Sports Items
	Storehouse	Screwdriver	Shave	Go-Chart
	Silo	Wrench	Nortug	Skateboard
	Plow	Hammer	Margrethe	Basket
	Hay on a rack	File	Rhianna	Bowling
	Threshing machine	Planer	Mette Marit	Table Tennis
	2. Flags	5. Constructions		
	Brazil	Lift		
	Spain	Snow plough		
	USA	Waste truck		
	Japan	Steamroller		
	Canada	Dump truck		
	3. Sea Related Items	6. Board Games		
	Mussels	Memory		
	Scallop	Playing cards		
	Glass jellyfish	Ludo		
	Jellyfish	Yahtzee		
	Crab	Uno		
P6	1. Planets	4. Boats	7. Board Games	8. Farm Related Items
	Venus	Skiff	Yahtzee	Silo
	Mars	Ferry	Chess	Threshing machine
	Pluto	Canoe	Ludo	Storehouse
	Saturn	Rib	Snakes & Ladders	Plow
	Mercury	Kayak	Uno	Barn
	2. Places in Vestfold county	5. School Related Items		
	Slottsjellet	Lunch Box		
	Tønsberg	Ruler		
	Sandefjord	Compass		
	The Hal of Memory	Backpack		
	Færder (lighthouse)	Gym bag		
	3. Birds	6. Vegetables		
	Bullfinch	Onion		
	Eagle	Beans		
	Peacock	Kohlrabi		
	Cockatoo	Cauliflower		
	Flamingo	Celery		
P7	1. Dog Breeds		4. Star Signs	5. Leaves
	Labrador	Sagittarius	Chestnut	Aspen
	Rottweiler	Libra	Oak	
	English setter	Aries	Roe	
	Norwegian moose dog	Taurus	Beech	
	Irish terrier		Capricorn	
	2. Gemstones			
	Sapphire			
	Grenade			
	Jade			
	Opal			
	Emerald			
	3. Pasta			
	Fusilli			
	Cellentani			
	Farfalle			
	Penne			
	Tagliatelle			
P 8	1. Places in Vestfold		4. Dog breeds	5. Gemstones
	Mølen	Border Collie	Emerald	Ruby
	The Hal of Memory	Labrador	Opal	
	Tjøme (The End of the World)	Rottweiler	Jade	
	Oseberg	Daks	Sapphire	
	Bastø		English setter	
	2. Flags			
	Brazil			
	China			
	Canada			
	USA			
	Spain			
	3. Planets			
	Venus			
	Mars			
	Pluto			
	Neptune			
	Jupiter			
P9	1. Flower	4. School Items	7. Sports Items	8. Gemstones
	Dandelions	Sharpener	Skateboard	Sapphire
	Tulip	Pencil case	Go-cart	Amethyst
	Clover	Compass	Basket	Ruby
	Lilac	Pencil	Table Tennis	Diamond
	Oxeye daisy	Ruler	Bowling	Pearle
	2. Sea Related Items	5. Bugs		
	Shrimp	Fly		
	Mussels	Beetle		
	Crab	Dragonfly		
	Jellyfish	Grasshopper		
	Glass jellyfish	Mosquito		
	3. Flags	6. Traffic Signs		
	Brazil	Tunnel		
	China	Highway		
	USA	Hospital		
	Sweden	Picnic area		
	Germany	Pedestrian crossing		

The number prior to each category indicates the number of the set and the sequence the sets were introduced

During data collection, we used prepared pencil-and-paper scoring sheets for each condition. A video camera was used in some sessions, when an observerer from the daycare center was not able during the sessions. The observers from the daycare centers were previously trianed to collect trial-by-trial data. After supervision by the first author, the observers from the daycare centers assessed relability and procedural fidelity.

### Dependent Measures

The primary dependent measure was the number of correct responses during all types of Inc-BiN probes, which included the total number of correct listener responses, tacts, and manded tacts. During each probe block we assessed responding to one stimulus set of five different pictures, which were presented four times each. All probe blocks consisted of one 20-trial block of the naming experience, followed by a 20-trial block of listener probes, a 20-trial block of tact probes, and a 20-trial block of manded tact probes. One block of baseline Inc-BiN probes––across three stimulus sets––consisted of 180 trials in which no programmed consequences followed. That is, each probe set included 60 trials (i.e., 20 tacts, 20 manded tacts, and 20 listener responses; each of the five stimuli in a set was probed four times each for tacts, manded tacts, and listener responses) in the absence of programmed differential consequences. The Inc-BiN probes are described in greater detail below.

A secondary dependent measure was the number of correct responses during MOI, in which four response types were rotated within each 20-trial block. Two different listener responses (selection responses during MTS and point to responses) and two types of speaker responses (tacts and manded tacts) were rotated from trial to trial. MOI trials are described in greater detail below.

### Data Collection

Frequency data were collected across all experimental conditions by hand, using prepared data-collection sheets. All trial blocks consisted of 20 trials and were recorded on a trial-by-trial basis. To be scored as correct, the response had to be emitted within 6 s of the onset of the discriminative stimulus. If no response occurred within 6 s after the instruction was presented or an incorrect response was emitted, the trial was scored as incorrect. During the naming experience (matching-to-sample tasks) and MOI blocks, a correct trial required that the participant responded correctly without any prompts.

### Experimental Design

**Fig. 1:**
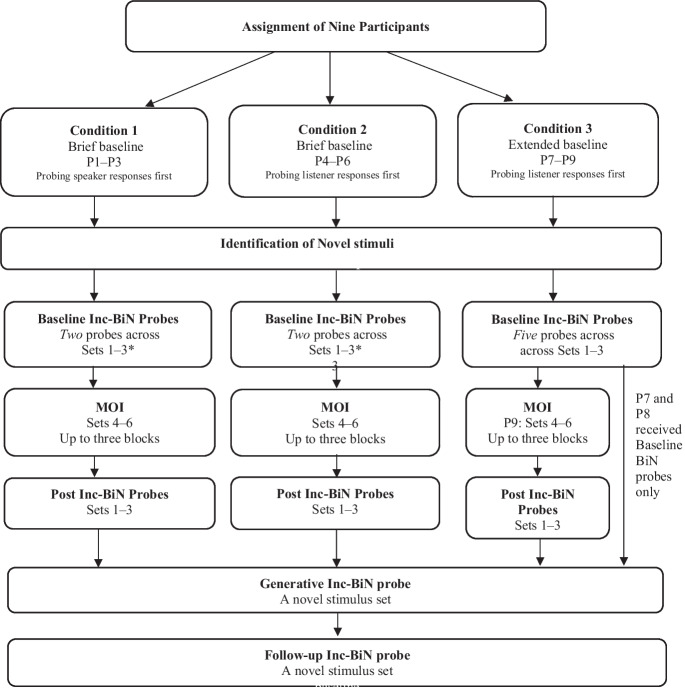
Overview of the experimental sequence. *Note. P* stands for participant and the numbers indicate an individual participant. P1–P3 were assigned to Condition 1, P4–P6 were assigned to Condition 2, and P7–P9 were assigned to Condition 3. * Indicate exceptions: P3 in Condition 1 received five baseline Inc-BiN probes and in Condition 2 P4 received three baseline Inc-BiN probes

The design was selected to enable a thorough analysis of the extent to which repeated MTS-naming experiences followed by Inc-BiN probes alone strengthened Inc-BiN skills, or alternatively, whether MOI was a critical independent variable. In the present experiment, we used a multiple probe design across three stimulus sets for each participant to answer the research questions. The design was set up to randomly assign nine participants to three different conditions––two brief-baseline conditions, Condition 1 and Condition 2, and one extended-baseline condition, Condition 3. Participants assigned to Condition 1 were designated P1, P2, and P3, while those assigned to Condition 2 were designated P4, P5, and P6, and those assigned to Condition 3 were P7, P8, and P9 (see Fig. 1).

Condition 1, a brief-baseline condition, consisted of probing speaker responses (tacts and manded tacts) before listener responses. Condition 2, a brief-baseline condition, consisted of probing listener before speaker responses. In Condition 3, an extended-baseline condition, listener responses were probed first.

After completion of baseline Inc-BiN probes, MOI was introduced for the participants who were randomly assigned to the brief-baseline conditions. The two brief-baseline conditions (Conditions 1 and 2) consisted of 2–3 baseline Inc-BiN probes, until a stable baseline was achieved before MOI was initiated. Baseline was considered stable if scores did not differ more than four responses within each set across baseline Inc-BiN probes. When responding met stability during baseline Inc-BiN probes for participants assigned to Conditions 1 and 2, MOI was initiated. If the stability criterion was not met, the participant would be exposed to additional baseline Inc-BiN probes, up to a maximum of five baseline Inc-BiN probes (as for P3), across Sets 1–3.

For the participants who were assigned to the extended-baseline condition (Condition 3), exposure to baseline Inc-BiN probes continued up to a maximum of five probes. For ethical reasons, the baseline was not extended without programmed consequences beyond the five baseline Inc-BiN probes. In Condition 3, if Inc-BiN skills did not meet the emergence criterion within the programmed number of baseline Inc-BiN probes, MOI was introduced (as for P9). Regardless of the length of the baseline, a maximum of three blocks of MOI (Sets 4–6) were trained.

### Pre-experimental Procedures

#### Preference Assessment

During the matching-to-sample tasks in the naming experience and MOI, preferred toys, edibles, or tablet games were used. Preferred stimuli were assessed using a multiple stimulus assessment without replacement procedure (DeLeon & Iwata, 1996). The preferred stimuli were delivered contingently on correct responses along with typical social consequences, such as enthusiastic praise, high fives, tickles, and smiles. 

#### Identification of Novel Stimuli

Tacts, manded tacts, and listener responses were assessed, in that order, for each pictorial stimulus. During identification of novel stimuli, MTS responses were not tested (see inclusion criteria above). Tacts were assessed by holding up the unknown stimulus in front of the participant and scored as novel if no responses or incorrect responses occurred within 6 s after a stimulus was presented. Manded tacts were tested in a similar manner, except that the vocal instruction “What is this?” was presented approximately 2 s before presenting the test stimulus. Listener trials were conducted by presenting five pictorial stimuli on the table in front of the participant and the participant was asked to point to one of them (e.g., “Point to the opal”). The response was considered correct if the participants pointed to the picture that corresponded with the instruction (e.g., the picture of the opal) within 6 s after the instruction was presented.

Each operant was tested three times with each of the five stimuli within a set. Thus, testing a stimulus set for novelty consisted of a total of 15 trials across the three operants. The stimulus was considered as novel if no correct tacts and manded tacts were emitted within 6 s following stimulus presentation, and a maximum of one correct listener response in the presence of the same stimulus. With five stimuli available for point-to responses, random listener responses could be emitted correctly by chance. Throughout the listener trials, all responses to a stimulus included in the probe sets had to be below chance (20%). If a correct listener response occurred more than once to a stimulus in a set during the identification of novel stimuli, it was used during MOI blocks. If more than one listener response or a correct tact response occurred to a stimulus, that particular stimulus was replaced by another novel stimulus.

Novel stimuli were identified under extinction meaning that no differential reinforcement was delivered contingent on correct responses. During identification of novel stimuli, as well as during all Inc-BiN probes, maintenance tasks were interspersed after every third to fifth trial to avoid fatigue and reduced responding to trials. The maintenance tasks were targets that the participants had previously mastered, and preferred toys along with praise were used as consequences. Examples of maintenance targets included pointing-to and tacting relatives, animals, body parts, tacting functions of everyday commonplace objects, and answering personal knowledge questions (e.g., “How old are you?”).

### Experimental Procedures

An overview of the experimental conditions is displayed in Fig. 1.

#### Baseline Inc-BiN Probes

The purpose of baseline Inc-BiN probes (Sets 1–3 stimuli) was to evaluate the effect of repeated probing and MOI. The Inc-BiN probes consisted of two subphases: (1) Naming experience, and (2) Inc-BiN probes––testing the emergence of untaught listener responses, tacts, and manded tacts (each subphase is described in detail below). In the present experiment, all the Inc-BiN probes were procedurally identical in that the naming experience was implemented first and then the Inc-BiN probes were conducted. This sequence of presenting the naming experience followed by Inc-BiN probes occurred for all probe types (i.e., baseline Inc-BiN probes, post-MOI Inc-BiN probes, generative Inc-BiN probes, and follow-up Inc-BiN probes).

##### Naming Experience 

During simultaneous (auditory + visual)-visual MTS, the experimenter placed the five comparison pictorial stimuli on the table in front of the participants. Then, the experimenter presented the participant with the sample picture card facing the participant, and presented the tact (e.g., “Okapi”). Next, the experimenter handed the sample picture card to the participant. The correct response was defined as placing the sample picture card on the positive comparison stimulus (i.e., a selection response). During the naming experience, the participants were exposed to each of the five novel stimuli and corresponding tacts four times within each set, which constituted one 20-trial block for each baseline set. Thus, the naming experience across Sets 1–3 consisted of at least 60 trials per set which were interspersed with play breaks and Inc-BiN probes (as described below). Correct selection responses during MTS trials were differentially reinforced with praise and items identified through the preference assessment.

If an incorrect selection response occurred during the naming experience (MTS), a correction procedure was implemented by re-presenting the trial and then prompting the correct selection response by pointing to the corresponding comparison. During the following trials, the prompt was faded using a 0–6 s progressive delay prompting procedure until independent responding occurred. If an incorrect response occurred, a 0-s prompt delay was presented on the next trial. Then the same trial was repeated with a 6-s time-delay. This sequence was repeated until a correct response occurred without a prompt within 6 s. During the naming experience, the mastery criterion was 18 of 20 (90%) correct MTS responses for two consecutive 20-trial blocks, or 20 of 20 (100%) accuracy for one 20-trial block. Between the naming experience and the Inc-BiN probes, the participants were given a play break for 10–15 min.

##### Inc-BiN Probes 

During Inc-BiN probes, the tacts, manded tacts, and listener responses were probed separately under extinction in 20 trial-blocks. Tacts and manded tacts were probed by presenting a novel stimulus on the table in front of the participant. In the case of manded tact trials, the instruction “What is this?” was presented 2 s after a picture was presented. A correct tact or manded tact was scored if the correct tact occurred within 6 s after the discriminative stimulus was presented. Listener trials were conducted by placing five pictorial stimuli on the table in front of the participant, who was then asked to point to one of them (e.g., “Point to the opal”). A correct response was scored if the participant pointed to the picture that corresponded with the instruction given by the experimenter.

The emergence of listener responses, tacts, and manded tacts were scored the same way as during identification of novel stimuli. The emergence criterion for the acquisition of Inc-BiN was a minimum of 14 out of 20 correct responses (70%; c.f. Morgan et al., 2021) for listener responses, tacts, and manded tacts.

##### Test Sequences

P1–P3 (Condition 1 participants) were exposed to tact probes first, then manded tacts and, finally, listener responses. The remaining six participants, P4–P9 (Conditions 2 and 3), were exposed to the test sequence probing listener responses before speaker responses (i.e., testing point-to responses, then tacts, and finally manded tacts).

#### Generative Inc-BiN Probe

The purpose of a generative Inc-BiN probe was to measure the extent to which Inc-BiN skills occurred using a novel stimulus set. All participants were exposed to a generative probe. The generative Inc-BiN probe was administered either after post-MOI Inc-BiN probes (as for P1–P6 and P9) or after the fifth baseline Inc-BiN probe (P7 and P8). The probing procedure was procedurally identical to the baseline Inc-BiN probes.

#### Follow-Up Inc-BiN Probe

For eight of the nine participants, a follow-up probe with another set of novel stimuli was conducted one month after the generative Inc-BiN probe to assess for maintenance of responding. For P2, the experiment ended before a follow-up probe was conducted because he left the daycare center to enter primary school. Follow-up Inc-BiN probes were procedurally similar to the baseline Inc-BiN probes.

#### Mixed-Operant Instruction (MOI)

During the 20-trial MOI blocks, the operants were trained until mastery in the following order: echoics and selection responses during MTS trials, tacts, manded tacts, and listener responses. Rather than training the four operants separately, the operants were intermixed across training trials within a trial block. Additionally, the stimuli within a set were rotated. For example, in the presence of the first stimulus in the set (e.g., an image of a sapphire), a selection response (i.e., listener response) and an echoic were trained during auditory/visual-visual MTS. Next, a tact was taught in the presence of the second stimulus (e.g., an image of a grenade), a manded tact in the presence of the third stimulus (e.g., an image of a jade), and finally, a listener response in the presence of the fourth stimulus (e.g., an image of an opal). On the fifth stimulus in the set (e.g., emerald), the sequence was reset, beginning with an MTS task with training echoic and selection response. In this way, the four operants were rotated across the five stimuli.

During MOI, the five stimuli within each training set was presented four times per operant, which constituted 20 trials per trial block. Thus, each operant was trained in four trials in each trial block. The mastery criterion was 18 of 20 (90%) accurate responses for two consecutive trial blocks, or 20 out of 20 (100%) correct responses in one trial block.

If Inc-BiN did not emerge during the first block of post-MOI Inc-BiN probes following the first MOI-set, a second set of MOI was introduced. Further, if Inc-BiN skills did not emerge during the second block of post-MOI Inc-BiN probes, following two sets of MOI, training with the third MOI-set was initiated.

##### Correction procedure 

First, echoics and selection responses were trained during (auditory + visual)-visual MTS tasks. In addition to a correct selection response, the participant was required to echo the researcher’s tact of the sample stimulus (e.g., saying “sapphire”) within 6 s of the vocal antecedent. If an incorrect selection response occurred during MTS, the prompt-fading procedure used during the naming experience was implemented. However, if the participant correctly selected the comparison stimulus without an echoic, we scored the trial as incorrect, and the researcher prompted the correct echoic in the next trial by saying the name of the relevant stimulus (e.g., said “sapphire”) and by withholding the visual sample stimulus until the participant emitted the correct echoic (e.g., saying “sapphire”). A correct echoic resulted in the presentation of the visual sample stimulus. Initially, the researcher used a 0-s prompt delay procedure. Next, the vocal echoic prompt and withholding of the sample stimulus were faded according to a progressive delay prompting procedure. The prompts were faded by increasing the delay from 0–6 s. However, if an echoic response did not occur after the researcher had withheld the stimulus for 6 s, the prompting sequence was re-implemented starting with a 0-s prompt delay. On the next trial, the experimenter modeled the correct response (i.e., the experimenter said the name of the sample stimulus and placed it on top of the identical comparison) and then said, “Your turn.” If the participant failed to emit the correct response within 6 s, the prompted trial was re-presented with an instruction (e.g., “Repeat after me, sapphire”) and the sample stimulus was withheld. A correct echoic (e.g., saying “sapphire”) within 6 s resulted in the presentation of the sample stimulus. Echoic prompts were faded by first fading the instruction (e.g., “Repeat after me…”) to merely uttering the initial syllables of the relevant words (e.g., “sa…”), followed by only the first sound (e.g., “s…”) and finally, all vocal prompts were faded.

Second, during tact trials, the experimenter held up the training stimulus in front of the participants. The correct response was to tact the stimulus within 6 s (e.g., saying “grenade”) of the presentation of the relevant stimulus. Third, during a manded tact trial (e.g., saying “jade”), a vocal antecedent was added (“What is this?”). During both types of tact trials, the correct response was saying the name of the stimulus presented. Novel tacts and manded tacts were initiated by providing an echoic prompt immediately following the presentation of the stimulus (i.e., a 0-s delay prompt). The echoic prompting procedure used to establish tacts was procedurally identical to the procedures described above to teach echoics during (auditory + visual)-visual MTS trials.

Finally, the pointing selection responses were taught during auditory-visual MTS. Stimuli were presented and instructions were provided in the same way as during the identification of novel stimuli, pre-experimental procedures, and during listener probes. Pointing or position prompts were used to teach listener responding. If an incorrect pointing response was emitted, the correct response was prompted on the following trials using a progressive prompt delay procedure. That is, correct pointing responses to the training stimuli were immediately prompted (0-s delay). Then, the researcher repeated the same trial by progressively expanding the delay between the antecedent and the prompt until the correct response occurred within 6 s. Across trained operants (selection response with echoic, tact, manded tact, and pointing responses), if three consecutive prompted trials did not produce a correct response, the researcher moved on to the next trial.

#### Post-MOI Inc-BiN Probes

After the mastery of an MOI-set, post-MOI Inc-BiN probes were conducted. These probes were procedurally identical to the baseline Inc-BiN probes, described above. Only participants who had received MOI were exposed to post-MOI Inc-BiN probes. These included all participants in Conditions 1 and 2. In Condition 3, P9 also received MOI, because five baseline Inc-BiN probes were not sufficient for speaker and listener responses to meet the mastery criterion. The same stimulus sets used during baseline Inc-BiN probes were used during post-MOI Inc-BiN probes.

### Interobserver Agreement

Trial-by-trial interobserver agreement (IOA) was scored from videotapes and in vivo by an independent observer during 55.9% (range 22–100%) of trial blocks selected randomly. In the present study, each procedural phase was assessed for IOA (i.e., identification of novel stimuli, the naming experience, Inc-BiN probes, and MOI trial blocks). All Inc-BiN probes (i.e., baseline Inc-BiN probes, post-MOI Inc-BiN probes, generative Inc-BiN probes, and follow-up Inc-BiN probes) and all MOI blocks were calculated together. The IOA was calculated as the number of trials with agreement divided by the total number of trials scored, multiplied by 100. The IOA criterion was at least 90% agreement. If the IOA scores fell below 90%, training and calibration between the two observers would be implemented. During identification of novel stimuli and the naming experience, the mean IOA was 100% agreement. During Inc-BiN probes the mean IOA was 99.7% agreement (range 98–100%). Finally, across MOI trial blocks IOA was 98.2% (range 95–100%). Across participants and conditions, the total agreement was 99.5% (range 96.6–100%).

### Procedural Fidelity

Procedural fidelity (PF) was collected for at least 30% of all trial blocks across all conditions and participants and was calculated by dividing the number of correctly implemented training components by the total number of training components and multiplying by 100. During identification of the novel stimuli phase, during tact trials, we assessed whether the stimuli were presented without any vocal supplements and that there was only one correct listener response emitted per stimulus. Additionally, during this phase, we also assessed whether the experimenter delivered reinforcement or implemented the prompting procedure. The PF during identification of novel stimuli was 100%. During the naming experience, we measured the extent to which the experimenter required only selection responses during MTS tasks and PF was 100%. During Inc-BiN probes we assessed whether the experimenter arranged an extinction condition with interspersed maintenance trials, and whether they implemented the correction procedure or provided consequences. Additionally, during Inc-BiN probes, we checked whether the experimenter used the programmed probe sequence. PF across Inc-BiN probes was 100%. During MOI, we assessed PF on intermixing of trial types within the trial blocks, that the correction procedure was used accurately for each operant, and that the relevant stimulus was presented during tact trials. For all seven participants who received MOI, PF across trial blocks was 100%. Finally, correct continuation criterion was examined for MTS (i.e., naming experience) and MOI trial blocks and that the criterion was held constant throughout the experiment.

Although the standard procedure during Inc-BiN probes included the interspersal of maintenance tasks on every third to fifth trial, the procedure was adjusted for P8 and P9 to include the interspersal of maintenance tasks on every second trial to increase motivation and responding to the experimental tasks. In addition, because of consistent incorrect responses to two specific stimuli (e.g., pencil case and ruler) by P4, P6, and P9, those two stimuli were trained in isolatation in one or two 20-trial blocks before being incorporated back into the MOI-trial blocks.

## Results

During the identification of novel stimuli, before probing and training, responding to the five stimuli in each of the 5–8 stimulus sets met the novelty criteria for all participants as depicted in Table 2. Before baseline Inc-BiN probe blocks were initiated, none of the participants engaged in tacts or manded tacts to the baseline Inc-BiN probe stimuli (Sets 1–3). The participants emitted a mean of 5.3% (range 0–23.3%) correct listener responses, which was below chance level.

**Table 2:** Results of the identification of novel stimuli

Participants	Number of correct tacts	Number of correct manded tacts	Number of correct listener responses	Number of stimuli included (sets)
P1 (BBM)	0/120	0/120	2/120 (1.6%)	**40 (8)**
P2 (BBM)	0/105	0/105	0/105 (0%)	**35 (7)**
P3 (EBM)	0/90	0/90	4/90 (4.4. %)	**30 (6)**
P4 (BBM)	0/120	0/120	28/120 (23.3%)	**40 (8)**
P5 (BBM)	0/120	0/120	7/120 (5.8%)	**40 (8)**
P6 (BBM)	0/120	0/120	2/120 (1.6%)	**40 (8)**
P7 (EB-O)	0/75	0/75	1/75 (1.3%)	**25 (5)**
P8 (EB-O)	0/75	0/75	0/75 (0%)	**25 (5)**
P9 (EBM)	0/120	0/120	12/120 (10%)	**40 (8)**

Each novel stimulus was tested in extinction, three times for each operant, until five stimuli in a set were obtained. Each operant was tested 15 times across stimuli in one set. Thus, maximum test trials per operant was 120 (15 test trials per operant across eight sets are 120 trials). *BBM* stands for brief baseline before mixed operant instruction, *EBM* stands for extended baseline before mixed operant instruction, *EB-O* stands for extended baseline only

During the naming experience before the baseline Inc-BiN probe blocks were conducted, all participants’ responding met the mastery criterion for selection responses during (auditory + visual)-visual MTS trials. Five of nine participants (P1, P4, P5, P7, and P8) mastered the MTS-responses immediately, and the remaining participants’ responding met the mastery criterion within two 20-trial blocks using 1–2 stimulus sets.

**Fig. 2:**
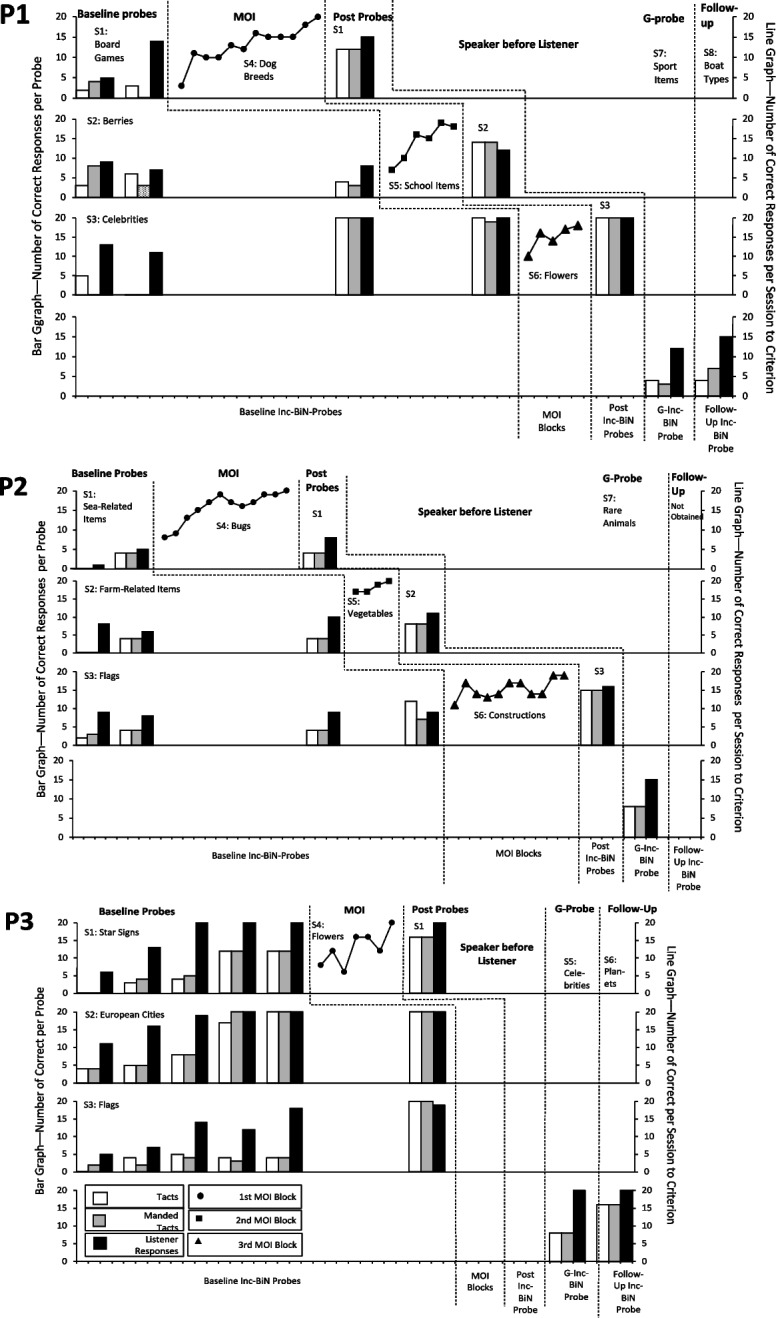
Results of Condition 1––Inc-BiN probes and MOI trial blocks––probing speaker first. *Note*. Fig. 2 demonstrates the number of correct responses in each Inc-BiN probe block and MOI block for participants assigned to Condition 1, a brief baseline followed by MOI, testing speaker before listener responses––P1, P2 and P3. *Inc-BiN* stands for incidental bidirectional naming, *S* stands for Set and *MOI *stands for Mixed-Operant Instruction. G stands for generative

**Fig. 3:**
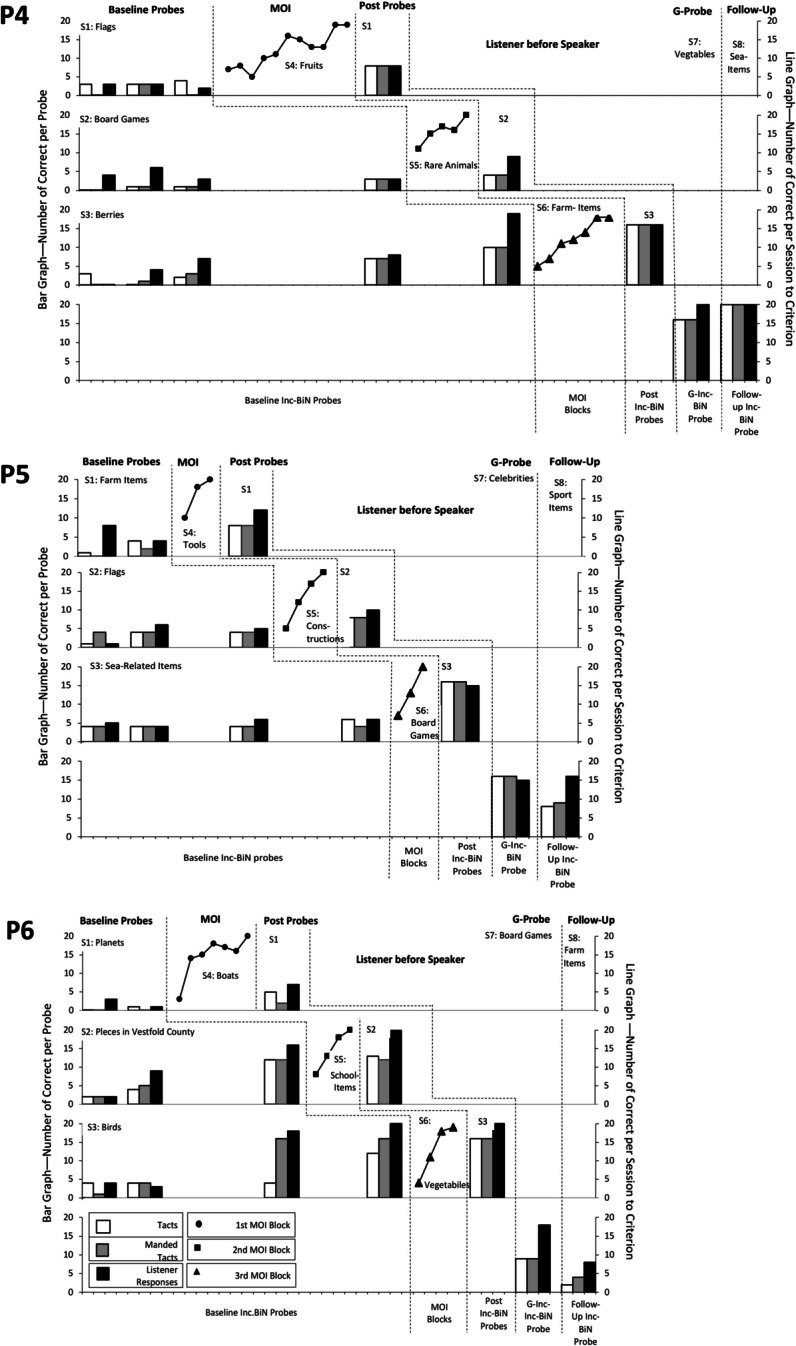
Results of Condition 2––Inc-BiN probes and MOI trial blocks––probing listener first. *Note.* Fig. 3 shows the number of correct responses in each Inc-BiN probe block and MOI block for participants assigned to Condition 2, a brief baseline followed by MOI, testing listener before speaker responses––P4, P5 and P6. *Inc-BiN* stands for incidental bidirectional naming, *S* stands for set and *MOI* stands for mixed-operant instruction, and *G* stands for generative

**Fig. 4:**
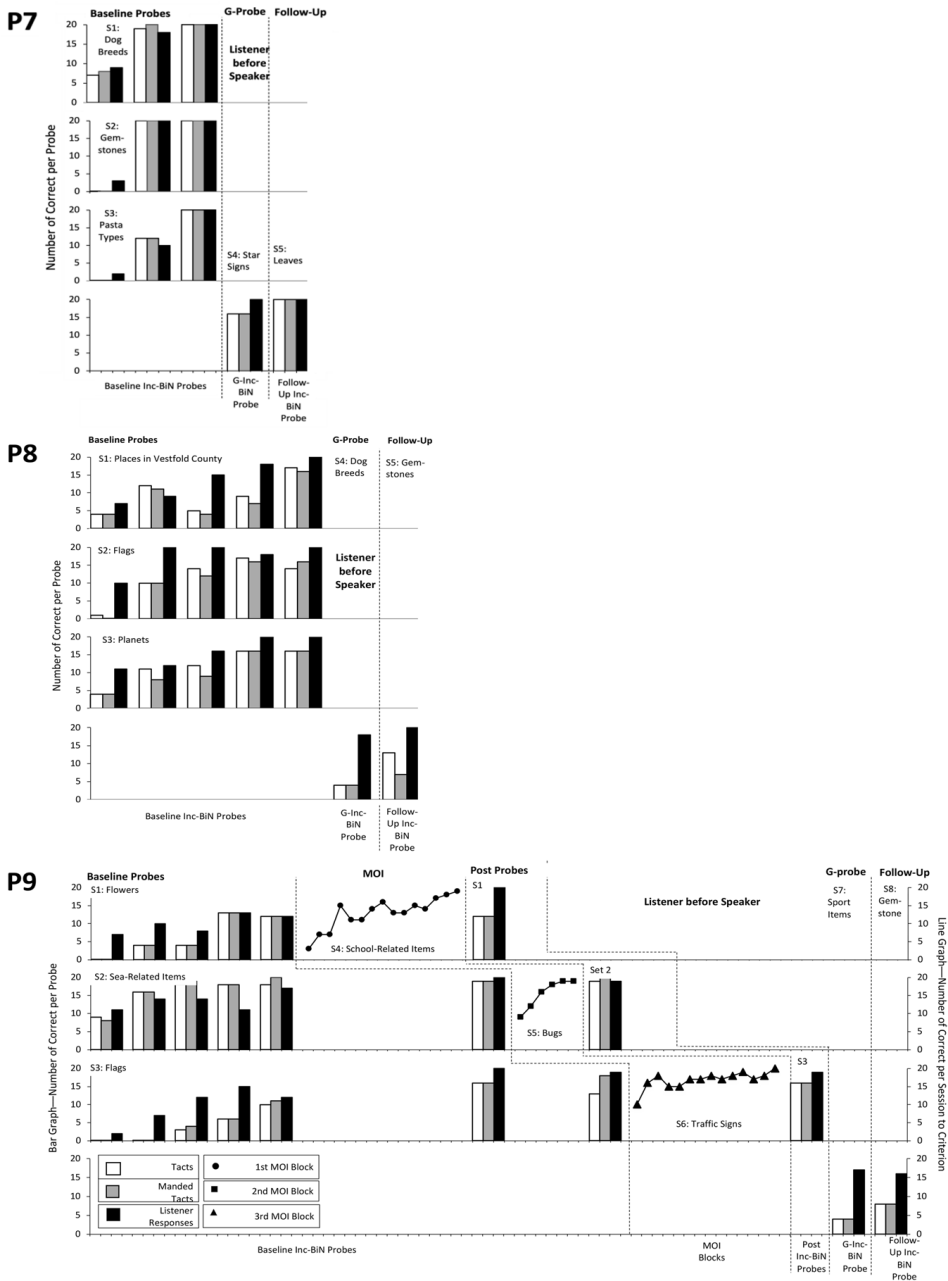
Results of Condition 3––Inc-BiN probes––probing listener first and MOI blocks for P9. *Note*. Fig. 4 illustrates the number of correct responses in each Inc-BiN probe block and MOI block for participants assigned to Condition 3, an extended baseline followed by MOI, testing listener before speaker responses––P7, P8 and P9. *Inc-BiN* stands for incidental bidirectional naming, *S* stands for Set and *MOI* stands for Mixed-Operant Instruction. G stands for generative

Figures 2, 3 and 4 show the percentage of untaught listener and speaker responses for each participant during the baseline Inc-BiN probe blocks, the post-MOI Inc-BiN probes, the generative Inc-BiN probes, and the follow-up Inc-BiN probes, as well as percentage of correct responses during each MOI block. The maximum number of responses in each experimental condition was always 20. Of the six participants assigned to the brief-baseline conditions (P1–P6), only P3 displayed substantial improvement in the number of correct responses during baseline Inc-BiN probes and was therefore exposed to additional baseline Inc-BiN probes, up to a maximum of five. Thus, P3 received an equal number of baseline Inc-BiN probes as the participants in Condition 3. Conditions 1 and 2 participants, excluding P3, P1–P6 demonstrated few Inc-BiN skills during the first baseline Inc-BiN probe block, as shown in Fig. 2 (Condition 1) and Fig. 3 (Condition 2). Across the first and the second baseline Inc-BiN probe blocks, for participants P1–P6 (excluding P3), listener responses occurred at a mean of 5.3 responses (range mean 2.3–10.6 responses; see Figs. 2 and 3). An average of 2.6 tact responses (range 0.6–4 responses), and an average of 2.3 manded tacts were emitted (range 0–4 responses). For the participants who experienced the brief-baseline conditions, the mean differences in the correct number of tacts from the first to the second baseline Inc-BiN probe block ranged between 0.6 and 3.3. Manded tacts varied from 0 to 4 correct responses and listener responses ranged from a mean of 1.3 to 9 correct responses across Sets 1–3. Overall, the mean improvement from the first to the second baseline Inc-BiN probe was 0.3–3.4 for tacts, 0.7–3.0 for manded tacts and 0–2.0 for listener responses. Thus, for five out of six participants exposed to the brief-baseline conditions, there was a minimal increase in listener responses and tacts, except for P3. P3, in Condition 1, was exposed to three more baseline Inc-BiN probes (a total of five baseline Inc-BiN probes; Fig. 2). After the second baseline Inc-BiN probe, P3 emitted a mean of 4.7 listener responses, which was above the stability criterion for the brief-baseline condition. From the second to the third baseline Inc-BiN probe, P3’s mean number of correct responses increased to 1.6 tacts, 2.0 manded tacts, and 5.7 listener responses.

Figure 4 displays the results for the participants who received an extended baseline with a maximum of five baseline Inc-BiN probes. Participants who experienced the extended-baseline condition also emitted few responses during the first baseline Inc-BiN probe block. During the first baseline probe block, P7–P9 emitted a mean of 2.8 tacts and 2.7 manded tacts (range 1.3–3.0 and 2.0–2.7, respectively), and a mean of 6.8 listener responses (range 4.6–9.7), which was similar to the numbers of correct responses emitted by the participants assigned to the brief-baseline conditions. For P7–P9, the mean number of correct responses improved from the first to the second Inc-BiN probe from 2.7 to 14.9 tacts, from 1.6 to 14.6 manded tacts, and from 3.7 to 15.4 listener responses.

Participants who were exposed to the extended-baseline condition showed improved Inc-BiN skills across the five baseline Inc-BiN probe blocks. Only P7 and P8’s responding met the emergence criterion for Inc-BiN after repetitious probing alone (Fig. 4), despite the low number of listener and speaker responses during the first baseline probe block. P7 already demonstrated Inc-BiN skills during the third baseline Inc-BiN probe block and was therefore not exposed to additional baseline Inc-BiN probes. The remaining participants in Condition 3 (P8 and P9) experienced all five baseline Inc-BiN probe blocks. For P9, however, Inc-BiN skills did not meet the emergence criterion after the five baseline Inc-BiN probes; therefore, MOI was initiated.

Seven of nine participants (P1, P2, P3, P4, P5, P6, and P9) experienced MOI after baseline Inc-BiN probe blocks. These participants were exposed to between one and fifteen MOI blocks (thus exposed to some or all of the training sets; Sets 4–6) before their responding met the criterion to move on to post-MOI probes, as shown in Fig. 2 for P1–P3, Fig. 3 for P4–P6, and in Fig. 4 for P9.

During the post-MOI probes, all seven participants (P1–P6 and P9), who received MOI, demonstrated increases in the number of speaker and listener responses from post-MOI probes of Set 1 stimuli to post-MOI probes of Set 2 and finally to post-MOI probes of Set 3 stimuli. P3 received only one block of MOI (see Fig. 2) before Inc-BiN skills reached the emergence criterion during the first post-MOI Inc-BiN probe across all probe sets (Sets 1–3). For the participants who experienced MOI trial blocks, the mean score on the last post-MOI Inc-BiN probe was 17.9 listener responses compared to a mean of 5.3 during the first baseline Inc-BiN probe, a mean of 17 tact responses compared to a mean of 2.6, and, finally, a mean of 17 manded tact responses compared to 2.3 during the first baseline Inc-BiN probe. Thus, considering mean scores during post-MOI probes, six of seven participants’ responding met the emergence criterion for the emission of Inc-BiN in the third and last post-MOI probe (see Figs. 2–4). Individual scores show that P1, P2, P4, P5, P6, and P9’s responding met the emergence criterion for Inc-BiN after three MOI sets during the post-MOI probes of Set 3 stimuli.

During the generative Inc-BiN probe block, responding for the six participants (P1–P6) exposed to the brief-baseline conditions met the criterion for the UniN (listener part of Inc-BiN) as shown in Figs. 2 and 3––the fourth panel for each participant. Furthermore, two of five participants’ (P4 and P5) responding met the criterion for both the speaker and the listener parts of Inc-BiN (Fig. 3, fourth panels), while one participant’s (P1) responding did not meet the mastery criterion for Inc-BiN (see Fig. 2). Among those participants who experienced Condition 1, a brief baseline with the probe sequence of speaker before listener, the three participants (P1–P3) demonstrated UniN, only. Although P3 experienced five baseline Inc-BiN probes and one MOI block, he did not achieve Inc-BiN skills within the mastery criterion during the generative Inc-BiN probe, only for UniN. In contrast, responding for all three participants (P4–P6) in Condition 2, which included a brief baseline with the probe sequence of listener before speaker, met the criterion for UniN and Inc-BiN emerged for two of them (P4 and P5).

Finally, during the generative Inc-BiN probes for the participants exposed to the extended-baseline condition (Condition 3; Fig. 4), all three (P7–P9) demonstrated UniN, while one of them (P7) emitted Inc-BiN to the emergence criterion. Thus, one out of three participants exposed to the extended-baseline condition achieved Inc-BiN (P7). Across all nine participants, the mean scores during the generative Inc-BiN probe were 10.6 tacts (range 4–16), 9.3 manded tacts (range 3–16), and 16.8 listener responses (range 12–20).

Follow-up Inc-BiN probes were conducted for eight of the nine participants. P2 (Condition 1) did not experience the follow-up Inc-BiN probe. The results are displayed in the fourth panel for each participant in Figs. 2, 3, and 4. The follow-up Inc-BiN probe demonstrated that four of the five participants (P1, P3, P4, and P5) in Conditions 1 and 2 maintained UniN (see Fig. 2 for P1 and P3 and Fig. 3 for P4 and P5). In the brief-baseline conditions, only P3 (Fig. 2) and P4 (Fig. 3) engaged in Inc-BiN skills to the mastery criterion. During the follow-up probe, one of the five participants (P6; Condition 2; Fig. 3) who was exposed to a brief-baseline condition, did not maintain the Inc-BiN that he acquired during post-MOI Inc-BiN probes.

During the follow-up Inc-BiN probes for the participants in the extended-baseline condition, all three participants (P7, P8 and P9) demonstrated UniN (see Fig. 4), whereas one of three participants (P7) maintained Inc-BiN skills within the emergence criterion.

## Discussion

### The Effect of Repeated Probing

The primary purpose of the present study was to investigate the degree to which repetitive naming experiences followed by baseline Inc-BiN probes might enhance Inc-BiN skills. The results showed that Inc-BiN improved in the three participants who were exposed to repetitious Inc-BiN probes (Condition 3). For two of them (P7 and P8), responding met the mastery criterion of Inc-BiN. Althrough, the Inc-BiN scores for the remaining participant (P9) continued to increase throughout the extended baseline condition, responding did not reach the emergence criterion within the five baseline Inc-BiN probe blocks. In addition, P3 (in Condition 1) was exposed to the same number of Inc-BiN probes as the participants in Condition 3. Althrough P3’s number of correct responses increased substantouly during the five baseline Inc-BiN probes, his responses did not meet the emergence criterion before MOI was initiated. However, for both P3 and P9, there is a possiblity that the Inc-BiN skills would have continued to improve if the baseline had been extended further.

Our findings support previous studies that have suggested that Inc-BiN can result from repeated naming experiences of exposure to novel object names (e.g., Byrne et al., 2014; Carnerero & Pèrez-Gonzàlez, 2014; Pérez-González et al., 2014; Petursdottir et al., 2020; Rosales et al., 2012; Solares & Fryling, 2019). In contrast to the present study, several MOI studies did not repeat the naming experience before post-MOI probes (e.g., Gilic & Greer, 2011; Greer et al., 2005, 2007; Longano & Greer, 2015). However, a recent study by Lee et al. (2021) evaluated two variations of post-intervention probes of Inc-BiN, one with and one without repeating the MTS-naming experience, which highlights the results of the present study. They found that the MTS-naming experience was a critical component for the post-MOI probes and demonstrated that Inc-BiN appeared within the criterion only when the MTS-naming experience was repeated before post-MOI probes (Lee et al., 2021). One possible mechanism that may help explain these results is that repeating the naming experience could facilitate participants’ echoing of the names spoken by the researcher. If echoics were present at the moment of reinforcement even if not explicitly programmed, the training of listener responses and tacts may have been trained directly (Schlinger & Blakely, 2024).

The results of the present experiment shed light on the findings from Longano and Greer (2015). Their results suggested that the MTS-naming experience produced more correct listener responses, tacts, and manded tacts during post probes of Inc-BiN than simply exposing the participants to novel tacts on a computer screen during joint attention. However, during both types of naming experience, they found a high number of echoics which likely occurred at the moment of reinforcement. If echoics were reinforced during the MTS-naming experience, while selecting the correct comparison stimulus (i.e., a listener response), both listener responses and tacts (i.e., the presence of the target stimulus while echoing its name) were likely directly trained to the test stimuli.

We did not measure echoic responses during the naming experience. However, all probing techniques, in one way or another, involved presenting vocal tacts in the presence of novel stimuli, mirroring the naming experience. Thus, measuring Inc-BiN through a naming experience followed by Inc-BiN probes may, unintentionally, have trained all Inc-BiN constituents: tacts, echoics, and listener responses.

Also, it is possible that specific pre-experimentally established skills can predict Inc-BiN outcomes of repeated probes. Specifically, both participants who demonstrated Inc-BiN according to the criterion in the extended-baseline condition (P7 and P8) were among those five who immediately mastered the selection responses during the MTS-naming experience. The spontaneous mastery of listener responses in the MTS-naming experience is likely due to a well-established echoic repertoire. P3, P7, and P8, who increased the number of correct listener and speaker responses substantially across the five baseline Inc-BiN probes, obtained scores in the domain of vocal imitation ranging from 93 to 100% on the ABLLS-R (see Appendix). However, P1 and P4 (in the brief-baseline conditions), who obtained the lowest scores on vocal imitation (52% and 85%, recpectively), were also among the participants who mastered the MTS-tasks during the naming experience in the first trial block. Hence, the exact impact of pre-experimental skills, such as the echoic, remains unclear.

### The Effect of Mixed-Operant Instruction

A second objective of this study was to investigate the extent to which MOI facilitated the acquisition of Inc-BiN when repetitive probes alone failed to do so. To demonstrate an increase in Inc-BiN skills as an effect of MOI, the same stimulus sets were used for both pre- and post-MOI probes. The results of these probes revealed that all six participants who were exposed to the two different brief-baseline conditions and subsequently to MOI, demonstrated increases in Inc-BiN skills. Likewise, the two participants who underwent the maximum number of five baseline Inc-BiN probes (P3 in Condition 1 and P9 in Condition 3) showed enhanced Inc-BiN skills before MOI commenced. For the five participants exposed to the brief-baseline condition (excluding P3), the improvement of Inc-BiN skills was observed after the MOI was introduced, not during the brief-baseline condition itself. These results replicate previous research that found MOI to be a successful intervention for the emergence of Inc-BiN (e.g., Byrne et al., 2014; Fiorile & Greer, 2007; Gilic & Greer, 2011; Greer et al., 2005, 2007; Hawkins et al., 2009; Olaff et al., 2017; Rosales et al., 2012). Consistent with previous research, MOI likely sped up or “boosted” the emergence of Inc-BiN skills and the reinforcement history across exemplars seemed to produce novel behaviors, such as the emergence of Inc-BiN skills (e.g., Sivaraman et al., 2023).

### The Impact of Probe Sequence

In the present study, probing listener responses first led to slightly more Inc-BiN skills than testing speaker responses first during post-MOI probes, generative Inc-BiN probes, and follow-up Inc-BiN probes. Although Inc-BiN skills were evident during post-MOI probes for the participants exposed to the probe sequence testing speaker first, only UniN was observed during generative Inc-BiN probes in two (P2 and P3) of the three participants in Condition 1. The third participant (P1) responded correctly to 12 of 20 listener trials. However, none of the three participants exposed to the sequence probing speaker first demonstrated Inc-BiN during the generative Inc-BiN probe. In contrast, responding for three of six participants (P4, P5 and P7) exposed to the probe sequence testing listener first during the generative Inc-BiN probe, met the criterion for Inc-BiN, while all of their responding met the criterion for UniN. Although all six participants’ responding demonstrated UniN during the generative Inc-BiN probe, independent of the probe sequence, the generativity of acquired Inc-BiN skills after MOI or repeated probing was modest in the present experiment and, thus, seems not to be affected by the probe sequence.

Also, the probe sequence did not impact the maintenance of the established skills. For all participants, regardless of probe sequence, the maintenance of Inc-BiN skills was sparse. During follow-up probes for the participants who were exposed to listener probes first, UniN was maintained for all of them, except for one participant (P6), while Inc-BiN was maintained in two of six participants (P4 and P7). In contrast, during the follow-up probe for the participants exposed to the sequence probing speaker responses first, P1 demonstrated UniN only, while P3 maintained Inc-BiN. P2 did not undergo a follow-up probe. Thus, the results were slightly in favor of testing listener responses before speaker responses, at least during generative Inc-BiN probes. No such difference was apparent during post-MOI probes for the participants in the brief-baseline conditions, where three participants were exposed to speaker trials first (Condition 1) and three of them (Condition 2) were exposed to listener first during all probes. Therefore, the results of the present experiment did not strongly support the assumption that testing listener before speaker responses enhances the speaker part of Inc-BiN.

### The Maintenance of Emerged Inc-BiN Skills

Our findings from a one-month follow-up Inc-BiN probe suggests limited maintenance of the improved Inc-BiN skills regardless of condition. In other words, whether the improvement of Inc-BiN was a result of repetitive Inc-BiN probing or MOI, the long-term effect was modest. To our knowledge, this study is the first to include follow-up Inc-BiN probes in MOI-research, as well as in studies involving exposure to tacts during MTS tasks (the naming experience) as an independent variable.

### Strengths of the Current Experiment

A strength of the present study is the multiple probe design across stimulus sets, where participants underwent 2–5 baseline Inc-BiN probes. Typically, Inc-BiN is measured before an intervention is implemented, referred to as the pretest. Although MOI was delayed until additional Inc-BiN probes were conducted, repeated probing did not significantly improve Inc-BiN from the first to the second Inc-BiN probe, except in P7. However, responding for all participants in the brief-baseline condition met stability in baseline within 2–3 probes. Achieving a stable baseline contributes to a more accurate interpretation of the intervention’s effect.

A second strength of the current study is the assessment of the participants’ pre-experimental skills. Despite relatively high ABLLS-R scores on the operants included in the Inc-BiN cusp, none of the nine participants showed Inc-BiN skills within the emergence criterion during the first baseline Inc-BiN probe block. Thus, none of the nine participants demonstrated Inc-BiN before the experiment was initiated. Although the participants in the extended-baseline condition and the participants in the brief-baseline conditions demonstrated similarly low scores during the first baseline probe, the mean number of correct responses increased more from the first to the second baseline Inc-BiN probe for the participants in the extended-baseline condition. However, during the fifth baseline probe, Inc-BiN scores substantially improved for four participants, the three participants assigned to the extended-baseline condition (Condition 3) and P3 in the brief-baseline condition (Conditon 1), and responding for two of them met the Inc-BiN mastery criterion. This result indicates that the participants likely learned the names of the stimuli during probing, possibly through echoics as suggested by Schlinger and Blakely (2024). All participants scored high in the vocal imitation domain of the ABLLS-R (range 52–100% mastery): The participants in the brief-baseline condition obtained an average score of 85.33% mastery (range 52–98%), compared with the participants in the extended-baseline condition who obtained a mean score of 95.33% mastery (range 93–100%). However, P3 in the brief-baseline condition, who received five baseline Inc-BiN probes because of increasing correct responses, did not contribute to the conclusion that participants in the extended-baseline condition (Condition 3) were more likely to demonstrate Inc-BiN without MOI than the participants in Conditions 1 and 2.

### Limitations of the Present Experiment

A limitation of the present study concerns the naming experience. Responding to visual stimuli during (auditory + visual)-visual MTS tasks was previously well-established, as visual-visual MTS was a prerequisite for participation in the present experiment (see Appendix A). Accordingly, the participants could respond correctly to the visual-visual MTS tasks without responding to the vocal tacts of the sample stimuli (auditory stimuli). Lack of responding to the tacts of the sample stimuli may be a product of a limited history with respect to responding to auditory stimuli during visual-visual MTS. The vocal tacts of the sample stimuli alongside the visual stimuli as the antecedents constituted compound stimuli. Restricted correct responses during Inc-BiN probes could reflect blocking of stimulus control (Kamin, 1969), if responding to the auditory stimuli (the tacts by the experimenter) was absent or limited during the naming experience as a result of previous discrimination training of visual stimulus control.

Alternatively, one stimulus dimension could hinder stimulus control by a second feature. That is, essential stimulus traits could have been overshadowed (Miles & Jenkins, 1973). For example, P1 emitted “berries” to all the different berries in the stimulus set (Set 2), possibly because all utterances of the stimuli ended with berries except for the Norwegian word for cloudberries, *multer* (spelled out in Norwegian). However, this study did not measure whether the participants responded to the vocal tacts of the sample stimuli during the naming experience by, for example, requiring overt echoing. In addition, during the Inc-BiN probes, in contrast to during the naming experience, responses were not prompted and differentially reinforced. Limited responding during the subsequent Inc-BiN probes could, therefore, at least in part, reflect the fact that prompts had not been completely faded out during training or that enthusiastic praise was no longer forthcoming.

### Implications of the Results of the Present Study

Despite these limitations, results from the present study have an important methodological implication related to repeated probing to strengthen Inc-BiN skills in some participants. One implication is to isolate the effect of a naming experience and a relevant intervention (e.g., MOI). We likely need to avoid (auditory + visual)-visual MTS-naming experiences before the standard Inc-BiN probe protocol is commenced. Therefore, it is necessary to explore other forms of naming experience before probing Inc-BiN to avoid potential intervention implications.

### Future Research

First, the role of echoics during the naming experience is not ruled out. Although some experiments have made efforts to analyze the role of the echoic in Inc-BiN (e.g., Miller et al., 2021; Petursdottir et al., 2014, 2020), there is still a scarcity of research on this topic. In one of these studies, Miller et al. (2021) found limited support for the presence of echoics on tact emergence (i.e., Inc-BiN).

Second, joint attention is suggested by some researchers (e.g., Catania, 2013; Horne & Lowe, 1996; Miguel, 2016) to be an important prerequisite for incidental learning of novel names of stimuli. Obviously, joint attention impacts Inc-BiN. For example, according to Baldwin (1993), children actively observe information regarding an object that may correspond with the speaker’s naming of a stimulus. Therefore, we support the suggestion by Gilmore et al. (2024) that overhearing the tact during joint attention (e.g., Akhtar, 2005) could set a condition in which typically developing children learn names of novel stimuli. During interactions between two other persons, the participants may overhear the tacting of a novel stimulus and, thus, establish a novel listener and speaker response out of this naming experience. However, we are not aware of behavioral studies investigating overhearing during joint attention as a naming experience to facilitate Inc-BiN.

Third, a consideration of the role of the motivating operation (Laraway et al., 2003) in the naming experience and how it impacts the reinforcement of observing responses (Wyckoff, 1952) is merited. Usually, the naming experience involves exposing the participant to novel stimuli that they may have no interest in (e.g., rare birds). Of course, such novel stimuli are used to improve experimental control and avoid a history with the test stimuli. However, using toys that are more interesting stimuli for the child will likely increase the interest in learning the names of the items. The experimenter may allow the child to choose among several novel items (e.g., toys) and the one the child selects, the researcher tacts. Contriving a motivating operation as part of the naming experience will resemble a more natural context in which Inc-BiN typically occurs.

Fourth, future research should investigate the role of responses emitted at the moment of reinforcement. Dugdale and Lowe (1990) suggested that participants below the age of four formed equivalence classes when they talked to themselves, while those who did not engage in self-talk while completing MTS-tasks did not demonstrate responding in accordance with stimulus equivalence. If echoics occur during the naming experience, then uttering the name of the object is, in effect, reinforced directly, even if not explicitly, in the presence of the object (Schlinger & Blakely, 2024).

Finally, although seven of eight participants maintained UniN in a follow-up probe one month after the last generative probe, the maintenance of Inc-BiN was moderate. This result highlights the need for further research on the long-term effect of interventions aimed at enhancing Inc-BiN skills. Also, additional research investigating the effects of test sequences during Inc-BiN probes is warranted.

## Summary

The main contribution of the present study is the finding that repetitive exposure to the MTS-naming experience before Inc-BiN probes may be sufficient to increase Inc-BiN skills. This experiment extended previous research by isolating the effect of repetitive baseline Inc-BiN probes from the effect of MOI. In addition, when repeated probing did not strengthen these skills, MOI effectively increased Inc-BiN skills. However, related to the long-term effect of repeated Inc-BiN probes and MOI to enhance the generativity of Inc-BiN skills are modest. Effective procedures to establish Inc-BiN skills are an essential contribution to the field of effective verbal behavior training for children with ASD, as well as promoting the generativity and retention of the established skills. Therefore, it is important to rely on reliable probing techniques to measure these skills. Improving Inc-BiN skills should be a significant target in all behavioral intervention programs for children with ASD, as soon as prerequisite skills are acquired.

## Data Availability

The data associated with the present paper is available in a locked achieve at OsloMet––Oslo Metropolitan University and can be accessed by contacting the first author.

## Appendix A

see Table 3

**Table 3:** Participant characteristics

Participants	P1	P2	P3	P4	P5	P6	P7	P8	P9
Diagnosis	ASD	ASD	ASD	LD	ASD	ASD	ASD	ASD	ID
Age	5.0	3.6	4.10	5.10	4.6	5.0	6.0	5.0	6.2
Bilingual	No	No	No	Yes	Yes	Yes	Yes	No	No
**% ABLLS-R scores in domains relevant for language development**									
C & R effectiveness	85	95	100	96	98	100	100	92	94
Visual performance	49	77	100	77	93	100	100	100	69
Receptive language	63	80	98	72	78	78	99	92	88
Motor imitation	40	88	99	76	84	83	99	100	83
Vocal imitation	52	98	93	85	92	92	93	100	93
Requests	58	70	96	89	68	64	96	79	70
Labeling	61	53	77	71	56	44	97	77	84
Intraverbals	2	13	81	48	22	39	83	30	60
Spontaneous vocalization	47	64	100	100	82	82	100	100	100
Syntax and grammar	32	95	82	63	27	57	82	66	63
Generalized responding	75	100	92	100	100	83	92	83	75

*ABLLS-R* Assessment of basic language and learning skills-revised, *ASD* stands for autism spectrum disorder, *LD* = language delays, *ID* = intellectual disability, and *C & R* cooperation and reinforcement
